# Indoor Air Contamination from Hazardous Waste Sites: Improving the Evidence Base for Decision-Making

**DOI:** 10.3390/ijerph121214960

**Published:** 2015-11-27

**Authors:** Jill Johnston, Jacqueline MacDonald Gibson

**Affiliations:** 1 Division of Environmental Health, Keck School of Medicine, University of Southern California, Los Angeles, CA 90089, USA; 2 Department of Environmental Sciences and Engineering, Gillings School of Global Public Health, University of North Carolina at Chapel Hill, Chapel Hill, NC 27599, USA; jackie.macdonald@unc.edu

**Keywords:** vapor intrusion, hazardous waste, indoor air quality, environmental decision-making, contaminated sites

## Abstract

At hazardous waste sites, volatile chemicals can migrate through groundwater and soil into buildings, a process known as vapor intrusion. Due to increasing recognition of vapor intrusion as a potential indoor air pollution source, in 2015 the U.S. Environmental Protection Agency (EPA) released a new vapor intrusion guidance document. The guidance specifies two conditions for demonstrating that remediation is needed: (1) proof of a vapor intrusion pathway; and (2) evidence that human health risks exceed established thresholds (for example, one excess cancer among 10,000 exposed people). However, the guidance lacks details on methods for demonstrating these conditions. We review current evidence suggesting that monitoring and modeling approaches commonly employed at vapor intrusion sites do not adequately characterize long-term exposure and in many cases may underestimate risks. On the basis of this evidence, we recommend specific approaches to monitoring and modeling to account for these uncertainties. We propose a value of information approach to integrate the lines of evidence at a site and determine if more information is needed before deciding whether the two conditions specified in the vapor intrusion guidance are satisfied. To facilitate data collection and decision-making, we recommend a multi-directional community engagement strategy and consideration of environment justice concerns.

## 1. Introduction

**Figure 1:**
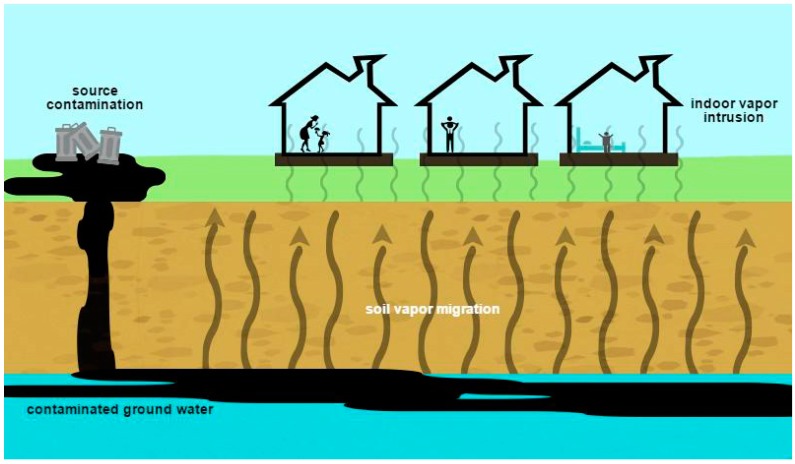
Schematic illustration of vapor intrusion pathway.

At sites where groundwater is contaminated with volatile chemicals, the chemicals can migrate through the overlying soil into buildings, contaminating indoor air (Figure 1). Such vapor intrusion is increasingly recognized as a major pathway for potential human exposure to contaminants at hazardous waste sites [1,2]. Some studies suggest that inhalation of vapors inside homes may be the most significant pathway by which communities are exposed to chlorinated solvents in groundwater contaminated by hazardous waste disposal sites [3,4,5,6]. Elevated rates of cancers, low birth weights, fetal growth restrictions, and cardiac defects have been reported at U.S. sites with chlorinated solvent vapor intrusion, although causality has not been confirmed [7,8].

The global magnitude of the vapor intrusion problem is unknown. A recent review suggested that groundwater is contaminated with toxic chemicals at “several hundred thousands of sites throughout the world” [9]. In the United States (U.S.) alone, groundwater at more than 120,000 waste sites has yet to be sufficiently cleaned up [10]. In addition, hazardous waste sites previously considered sufficiently cleaned are being re-evaluated to investigate the vapor intrusion pathway [11], and the U.S. Agency for Toxic Substances and Disease Registry is increasingly investigating public health concerns at vapor intrusion sites [2].

Reflecting the increasing recognition of vapor intrusion as a potential source of indoor air pollution near hazardous waste sites, in 2015 the U.S. Environmental Protection Agency (EPA) released a new guidance document on vapor intrusion, *Technical Guide for Assessing and Mitigating the Vapor Intrusion Pathway from Subsurface Vapor Sources to Indoor Air*. The guidance document calls for remediation of vapor-phase risks when: (a) the existence of vapor intrusion as an exposure pathway has been confirmed; and (b) either lifetime excess cancer risk is above a target threshold or exposure concentrations are above levels thought to increase risk for non-cancer health effects. The guidance document also calls for public involvement in the decision-making process concerning vapor intrusion remediation and notes this is especially important for such sites due to the potential impact on people’s personal property (their homes).

Although the U.S. EPA’s new policy guidance is clear that remediation is required when the vapor intrusion pathway is confirmed and risks are sufficiently high, substantial uncertainty exists in the evidence needed to demonstrate the existence or non-existence of an exposure pathway and the magnitude of risks where a pathway is confirmed. For example, the guidance does not state how many indoor air samples are needed to confirm whether or not an exposure pathway exists. Similarly, guidance on the number and duration of samples or the types of models to be used to estimate exposure concentrations for risk assessment is lacking. While the guidance emphasizes that multiple lines of evidence are needed to confirm or disprove vapor intrusion, it says little about how to integrate potential conflicting evidence sources (for example, one source suggesting the existence of a pathway and another suggesting the pathway is incomplete). In addition, the document does not offer suggestions for engaging community members in the process of collecting samples inside their homes or interpreting the various lines of evidence.

In this paper, we briefly describe the key decision-making concepts in the recent EPA vapor intrusion guidance document, focusing on the decision about whether remediation is necessary. Then, we review potential challenges to making remediation decisions that may not be adequately addressed in the guidance. We next present a method from the field of decision analysis that could help overcome some of these challenges, and we offer approaches for community engagement in the site assessment process in order to strengthen decision-making. Finally, we conclude with a summary of a decision-making process that could help address uncertainties and limitations of current decision-making at vapor intrusion sites. We hope the suggestions offered here will help implement the EPA guidance document and perhaps can be considered in future updates. In addition, approaches suggested here may be useful at vapor intrusion sites in other parts of the world.

## 2. To Remediate or Not to Remediate?

The EPA vapor intrusion guidance document states that vapor intrusion sites should be cleaned up when the vapor exposure pathway is complete and the human health risk is above agency target levels. The agency specifies, the vapor intrusion pathway “is referred to as ‘complete’ for a specific building or collection of buildings when the following five conditions are met.

(1). a subsurface source of vapor-forming chemicals is present underneath or near the building(s);
(2). vapors form and have a route along which to migrate (be transported) toward the building(s);
(3). the buildings are susceptible to soil gas entry, which means openings exist for the vapors to enter the building and driving “forces” exist to draw the vapors from the subsurface through the openings into the building(s);
(4). one or more vapor-forming chemicals comprising the subsurface vapor source(s) is (or are) present in the indoor environment; and
(5). the building is occupied by one or more individuals when the vapor-forming chemical(s) is (or are) present indoors.”

If the vapor intrusion pathway is deemed complete, the guidance outlines methods to conduct a human health risk assessment to determine whether the risk exceeds acceptable levels. The guidance explains that in determining whether cancer risk levels are acceptable, “EPA generally uses a cancer risk range of 10^−6^ to 10^−4^ as a ‘target range’ within which to manage human health risk as part of site cleanup”—in other words, no cleanup is needed if the contamination is expected to cause fewer than the equivalent of one extra cancer death per 10,000 to one million exposed people. For non-cancer risks, the guidance explains, “EPA generally recommends that the target HQ (hazard quotient) or HI (hazard index) not exceed 1”—in other words, that the ratio of the indoor air concentration to the concentration expected to cause non-cancer effects is less than one.

While the guidance states clearly the steps required to demonstrate completeness of the vapor intrusion pathway and the levels of risk deemed acceptable, implementing the fourth of the above steps (confirming whether or not vapors from the subsurface contamination are present in a given building) and quantifying exposure in order to estimate risks will be challenging. Step 4 is substantially complicated by the potential for extreme spatiotemporal variability in indoor air pollutant concentrations arising from vapor intrusion, which we further elaborate below. The risk assessment step is complicated both by this spatiotemporal variability and by uncertainties in modeling exposure concentrations at vapor intrusion sites—necessary for estimating risks at unmonitored locations and for projecting future risks if, for example, groundwater concentrations change or new buildings are constructed. These complexities are also described below.

## 3. Monitoring Vapor Intrusion

A critical component of establishing a complete vapor intrusion pathway is demonstrating the presence of the contaminant indoors (step 4 above). While this step may appear to be straightforward, there are many complexities to confirm the presence of a compound due to vapor intrusion. First, robust sampling methods are required because the concentrations of concern tend to be near the limit of detection for many common sampling techniques. Second, the concentration can change over space and time from nondetectable to spikes in concentration above health-based thresholds [12,13]. The EPA guidance recommends the use of time-integrated samples to confirm the presence of the chemical of concern, but the duration of time over which samples should be collected is not specified. Multiple sampling events are suggested to account for seasonal variability, but no specifics are offered. One could easily infer from the guidance that two samples are sufficient: the guidance states (p. 93), “For a typical-size residential building or a commercial building less than 1500 square feet, EPA recommends that the site teams generally collect one time-integrated sample in the area directly above the foundation floor (basement or crawl space) and one from the first floor living or occupied area, at least for the initial sampling round.” The required durations of these samples are not specified, but the guidance states that 24 h may be sufficient (p. 91): “Typically, for vapor intrusion investigations, indoor air samples are collected using six-liter canisters using sub-atmospheric pressure sampling over a 24-h period in residences or over an 8-h period (or workday equivalent) in commercial and industrial settings.” The number of buildings that should be sampled in a given community and the process for selecting buildings for sampling are not clearly articulated.

Existing evidence demonstrates that it is difficult to know which buildings will be impacted by vapor intrusion and at what level. Typically, the buildings affected by vapor intrusion sit atop the contaminated plume; however, the contamination attributable to vapor intrusion in one house does not adequately predict the concentrations in the house next door [14]. House-to-house variability (due to differences in construction styles and ventilation rates) can contribute up to two orders of magnitude to the variability in actual indoor air concentrations [15]. As a result, if indoor air monitoring is required to confirm the vapor intrusion pathway and assign human health risk, then a building-by-building analysis is needed to individually assess exposure. However, there is no mandate to test every building, so some areas may receive testing while others are left out. Priorities of monitoring may reflect the relative political power of residents or neighborhoods.

The body of evidence showing the heterogeneity of vapor intrusion exposures across not only space but also time is growing. Seasonal patterns, such as temperature, rainfall, pressure, and wind likely play some part in the temporal variability, although clear relationships have yet to emerge. One analysis of large datasets from two vapor intrusion sites showed considerable variability in short-term indoor air concentrations month to month and season to season [14]. A small longitudinal study showed 1–2 orders of magnitude fluctuation over the course of a month [13]. In one highly studied house, concentrations attributable to vapor intrusion varied on a daily basis, and over the course of two years the concentrations fluctuated up to three orders of magnitude [12]. In these three cases, the time period of maximum exposure was different. These results suggest that predicting the “worst-case” scenario is difficult.

As noted above, the vapor intrusion guidance document does not specify the extent or frequency of sampling needed to assess spatiotemporal variability in vapor intrusion risks. Portions of the guidance suggest that continuing the prevailing practice of collecting one to two 24-h samples may be acceptable for assigning exposure levels. The extrapolation of these results to long-term exposure scenarios is problematic. Previous research has concluded that the use of infrequent simplistic sampling schemes has a high potential of false negatives and provides poor information about long-term exposures [12,13,16]. In a heavily studied house affected by vapor intrusion, Holton *et al.*, concluded that if two samples were collected (one summer, one winter) there is only a 41% chance that one sample and 0% that both samples would exceed a health-based target if the target is equal to the long-term mean. In other words, in such a case, the false negative rate if only one sample is collected would be 59%. One the other hand, a sampling period that is too long may mask concern of high exposure periods [12], which may be of particular concern to vulnerable populations such as pregnant women.

Indoor air sampling for vapor intrusion is further complicated by the potential for confounding indoor or other non-vapor intrusion sources, as highlighted extensively in the EPA guidance [17,18,19,20]. Consumer products can off-gas the same chemicals of concern, introducing a different indoor source. Removing potential indoor sources can strengthen the evidence for vapor intrusion. The EPA recommends the identification and removal of confounding sources as well as assessment of the contribution of ambient sources. However, the process of removing indoor sources is inexact (due to the multitude of potential sources), time-consuming, intrusive (due, for example, to the requirement that homeowners remove cleaning products, dry-cleaned laundry, and other potential indoor sources for the duration of sampling), and potentially costly (for example, if costly real-time monitoring equipment is needed to detect indoor sources). One method, as suggested by some researchers, to help minimize the importance of indoor air sources is use of sub-slab vapor measurements, that is, analysis of the vapor directly beneath the foundation [21]. While sub-slab samples can serve as another line of evidence of a vapor intrusion pathway within the EPA guidance, these samples do not characterize actual exposure concentrations in the breathing zone of humans. A recent review of a large EPA database from multiple vapor intrusion sites suggests that in most scenarios the sub-slab vapor concentration is independent of the indoor air concentration and may only serve as a useful surrogate when the sub-slab concentration exceed 500 ug/m^3^ [22]. In homes with basements or a crawl space, these relationships may differ. Further, the potential spatial and temporal variability of sub-slab measurements is not well described.

Recent scientific publications offer guidance on potential ways to address the problems of spatiotemporal variability when deciding whether the vapor intrusion pathway is complete. Holton and colleagues (2013b) conclude that a 3-week sampling period at least quarterly would, in most cases, provide a reasonable estimate of the long-term exposure averages. Such specific recommendations of appropriate sampling plans would improve the utility of the EPA guidance.

Another potential solution is the use of novel, portable monitors that can collect near-real-time data on contaminant levels. Efforts are under way to develop small portable sensors to measure ambient chlorinated volatile organic compounds that may be adaptable to vapor intrusion [23,24,25]. Research into portable and inexpensive devices to collect vapor intrusion data has the potential not only to facilitate the collection of exposure data but also to enable community residents to participate in the exposure assessment process. These devices can collect detailed information to characterize temporal variability, offer real-time readings to residents, and store data electronically.

An additional potential solution is to use a controlled pressure method for step 4 of the vapor intrusion assessment process (determining whether groundwater chemicals are present indoors). This approach involves manipulating the indoor-outdoor pressure conditions to induce conditions for vapor intrusion (*i.e.*, under-pressurization) [16]. Recent studies indicate that this approach can eliminate false-negative readings from vapor intrusion, is less prone to temporal variability, and induces indoor air concentrations that typically exceed long-term average concentrations due to vapor intrusion [16,26]. Such an approach is still limited by the need for trained personnel and a house-by-house analysis to measure exposure, but it nonetheless offers a potential new tool to more quickly estimate potential indoor air concentrations at the household level.

## 4. Modeling Vapor Intrusion

The vapor intrusion guidance states, “When suitably constructed, documented, and verified, mathematical models can provide an acceptable line of evidence supporting risk management decisions pertaining to vapor intrusion” (p. 113). The guidance notes that models are especially useful when indoor air monitoring is infeasible. For example, models are the only way to project future risks in not-yet built structures or risk under alternative remediation scenarios. In addition, the EPA guidance document indicates that where indoor air monitoring is not possible (whether in current buildings or potential future structures) or for preliminary analyses, generic “attenuation factors,” which describe the proportional decrease in concentration in the indoor air compared to the groundwater or soil gas, can be used to predict indoor air chemical concentrations on the basis of measured groundwater or soil gas concentrations. There are a number of challenges associated with modeling, whether via generic attenuation factors or more complex models, not clearly addressed in the guidance.

The EPA guidance provides generic attenuation factors that it recommends as suitable for deciding whether indoor air concentrations are or are not sufficiently high to exceed the risk levels the agency has defined as acceptable. For the vast majority of sites except those with fine-grained soils, water tables less than five feet below the foundation, or known preferential pathways for vapor transport, the guidance recommends using an attenuation factor of 1/1000: that is, multiplying the soil gas concentration in equilibrium with the measured groundwater concentration (computed with Henry’s Law) by 1/1000, in order to estimate the indoor air concentration. For sites with “laterally extensive layers” of fine-grained soils, the agency recommends using an attenuation factor of 1/10,000. This approach is simple to implement because groundwater concentrations are typically already characterized at hazardous waste sites, so additional data are not required if using generic screening levels. However, research has demonstrated that groundwater concentrations are not adequate surrogates for measuring vapor intrusion exposure potential because variability in soil and household characteristics can lead to houses above relatively low groundwater concentrations having higher levels in indoor air than homes overlying higher concentrations and vice versa [14,27,28]. Furthermore, prior research using the EPA vapor intrusion database has shown that the current generic groundwater screening criteria may underestimate actual indoor air concentrations in a non-trivial number (>10%) of cases [22,27].

In an effort to represent site-specific conditions and in the absence of sufficient indoor air concentration measurements, many vapor intrusion site assessors use a model known as the Johnson-Ettinger model to predict indoor air concentrations [29]. This model is a one-dimensional fate and transport model that integrates information about the soil properties, foundation type and building conditions [30]. Prior research has suggested that with reasonable input parameters, the Johnson-Ettinger model can predict indoor air concentrations within one order of magnitude [15]. However, like the use of a generic attenuation factor, this model can underestimate actual indoor air exposure and thus fail to provide adequate protection to affected households [14,31,32,33,34]. Some researchers have cautioned against the use of this model because of the potential for false negatives and the frequency of under-predictions of measured exposure levels [35]. Alternatives to the Johnson-Ettinger model have been developed over the years, but these typically require detailed site- and house-specific inputs that may be difficult to collect; even with additional data, models to date typically are unable to adequately explain observations [36]. While complex three-dimensional models may be more accurate for an individual home, these approaches have not yet been scalable to a community level [37,38,39,40].

Despite its limitations, modeling is likely to be important for prioritizing sampling and predicting exposure concentrations due to the large number of homes typically present at vapor intrusion sites. The integration of probabilistic modeling into exposure assessment can address some of the limitations of both sampling and modeling by providing estimates of the potential range of indoor air concentrations a community may experience [33,34,41]. For example, we previously demonstrated that use of a multivariate statistical approach including variables describing soil type, groundwater depth, foundation, and season decreased the false negative rate, compared to using the EPA’s generic 1/1000 attenuation factor [27]. We also showed that a probabilistic version of the Johnson-Ettinger model, in which key inputs were represented as random variables, produced mean estimates that better predicted measured indoor air concentrations than when using the model in deterministic mode, although mean values from this model still tended to under-predict concentrations; we suggested using the 95th percentile values of the probabilistic model in order to produce more conservative exposure estimates [34].

Integrating modeling with measured data can substantially increase the utility of vapor intrusion models. In prior research, we showed that it is possible to calibrate a stochastic version of the Johnson-Ettinger model based on limited indoor air data to improve the predictions of indoor air quality at a community scale and to quantify the uncertainty [42]. This statistical approach generates a posterior range of indoor air concentrations that accounts for both the uncertainty about the parameters and the uncertainty that remains when some parameters are known [43], creating a more realistic picture to support decision-making [44]. Further, such an approach is not static, but rather facilitates the integration of new information, allowing an adaptability that is often seen as a desirable component of environmental policy tools. If new data are collected or a new understanding of important parameters or transport mechanisms emerges, previous analyses and data can be incorporated as prior knowledge through this method. Using available data (source concentration, indoor air measurements, *etc.*) with simulation and Bayesian calibration techniques allows for the explicit quantification of uncertainty in modeled predictions or screening exercises, which is crucial for meaningful interpretation of model results [42,45].

In summary, models are important at vapor intrusion sites because of the potential number of structures affected and the need to make timely assessments. Modeling exposure at vapor intrusion sites should adequately characterize the current state of evidence, its limitations and implications of these limitations. Inadequate articulation of uncertainties in environmental decision-making has contributed to inappropriate decisions and significant environmental and health damages [46,47]. Better understanding of uncertainty and how the level of uncertainty influences action is a prerequisite for better decision-making [48,49]. Because any site-level vapor intrusion assessments will be faced with imperfect information, and because modeling efforts are still advancing, the results of vapor intrusion exposure assessments need to be framed by confidence intervals. Integrating stochastic modeling methods into the site assessment process can advance understanding of the potential exposures across an entire community.

## 5. Integrating Multiple Lines of Evidence

The preceding review offers convincing evidence that the vapor intrusion pathway is highly complex, insufficiently described by current mechanistic models, and not well suited for application of simple decision heuristics such as the screening approaches described in the EPA guidance. Regulatory approaches that support environmental management decisions based on a single measured or predicted value are inadequate. It is important that the uncertainties of both indoor air measurements and modeled values are both acknowledged and quantified. Value-of-information approaches offer a possible framework for integrating multiple lines of evidence into the decision process in the face of the substantial uncertainties associated with vapor intrusion site assessment.

Value of information (VOI) analysis is a decision analytic technique developed to quantify the benefits of collecting additional information to reduce uncertainty before making a decision [50,51]. Broadly, VOI calculates the difference between the expected value of a decision made with currently available information and the expected value of a decision made after new information is gathered. Due to advances in computing technology, VOI’s use in medical and environmental health decision-making has grown substantially since the late 1980s [52,53]. Both the Presidential/Congressional Commission on Risk Assessment and Risk Management and the U.S. National Academy of Sciences have recommended using VOI techniques to improve environmental risk management decisions [53]. However, this approach is not mentioned in the EPA guidance document and has not been previously applied at vapor intrusion sites. Here we offer a stylized example of how VOI analysis could be used to help decide whether to collect additional lines of evidence before proceeding with a remediation decision at a vapor intrusion site.

For the example, consider a site where available lines of evidence leave the decision-maker uncertain about whether or not vapor intrusion is occurring in a specific home. Half the prior evidence suggests that vapor intrusion is active, while the other half does not, so that the decision-maker’s prior probability of vapor intrusion occurrence is 50%. The hypothetical cost to install a household remediation system (depressurization system or vapor barrier) is $1400. If vapor intrusion is occurring without remediation, then each of the four household residents faces an increased lifetime excess cancer risk of one in 10,000 (10^−4^). According to EPA policy, the monetary value of a premature death should be assigned a value of $7.4 million for cost-benefit analysis [54]. Therefore, if vapor intrusion exposure does in fact cause each household member to develop cancer and die prematurely, then the total health cost is $29.6 million. The decision-makers can decide whether this home should receive a vapor intrusion remediation system now, or they can collect more information before deciding. The sampling protocol under consideration has a false positive rate of 5% and a false negative rate of 20%. The decision-maker wants to know how much she should be willing to spend on this sampling protocol, if any, before making a remediation decision.

**Figure 2:**
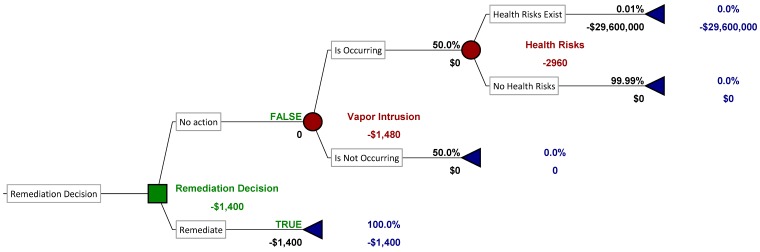
Decision tree for a hypothetical case in which the decision about whether or not to install a vapor intrusion remediation system for a home must be made on the basis of existing information. In this hypothetical case, existing information on the presence or absence of a vapor intrusion pathway into the home is inconclusive, so the chance that vapor intrusion is occurring is 50%. The hypothetical remediation system costs $1400 (shown on the lower branch). There is a 10^−4^ probability that the four members of the household will develop cancer, costing $29.6 million in lost life years, if vapor intrusion is occurring and no remediation system is installed. With no additional information, the best choice from an expected value maximization perspective is to install the remediation system.

Figure 2 illustrates the decision-maker’s choice in the absence of additional information. The square nodes represent decisions, the circular nodes chance events, and the triangles final outcomes. The costs at each stage are shown beneath each branch, and the probabilities of chance events as percentages above the branches. There are two chance events: whether or not vapor intrusion is occurring, with each outcome having an equal probability of 50%, and whether or not health effects will occur, assuming vapor intrusion is active. The chance of health effects is 10^−4^, or 0.01%. Using techniques describe elsewhere [50], this tree can be solved to calculate the expected value of each possible decision. In the absence of additional information, the decision to remediate costs $1400. The decision to take no action costs $1480 (in expected health costs). Therefore, if the choice is to be based on maximizing expected value (equivalent to minimizing costs), then the remediation decision is preferred. The expected value of the decision in this case is −$1400, which is the cost of the preferred option (remediation).

**Figure 3:**
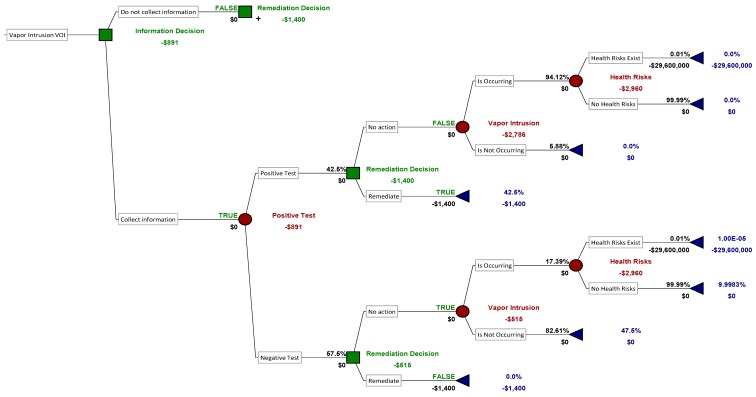
Tree representing the decision about whether to collect additional monitoring data before deciding whether to install the vapor intrusion remediation system for the hypothetical home under the scenario described in Figure 2. In order to provide value, this tree shows that the additional monitoring must cost less than $509, which is the difference between the expected value of the decision without collecting additional data (−$1400) and the value if additional data are collected (−$891).

Figure 3 illustrates how the decision problem changes if the decision-maker chooses to gather additional information. In this case, the first event in the tree (moving from left to right) is the result of the additional testing. The additional tests could be positive, suggesting that vapor intrusion is occurring, or negative. From the prior estimate of vapor intrusion occurrence (50%) and the information about the test’s false positive and false negative rates, Bayes’ Theorem can be used to calculate the total probability of a positive or negative test. The probability of a positive test is 42.5%, shown above the first branch on the tree, and the probability of a negative text is, correspondingly, 57.5%. The choice of whether to implement the remediation occurs after the decision-maker receives the test result. The probability that vapor intrusion is occurring now has changed, due to the new information. Again, Bayes’ Theorem can be used to calculate updated probabilities of vapor intrusion occurrence, given the test result. For example, if the test is positive, then the probability of vapor intrusion occurrence is updated from its initial value of 50% to 94%, shown along the upper chain of events on the decision tree. With the new information, the expected cost of the no-action decision increases from the initial estimate of $1480 to $2960, so remediation is still the preferred option. On the other hand, if the test is negative, then the expected cost of the no-action decision is $515, whereas remediation costs $1400, so the no-action option becomes the preferred option if using an expected value decision framework.

The value of the additional information can be calculated from the results of Figure 2 and Figure 3. In the absence of information, the decision-maker would choose to remediate, and the expected cost of this decision is $1400. However, once the additional information is gathered, then the expected cost becomes $891 (shown in Figure 2), a decrease of $509 compared to the expected cost of the decision without information. Therefore, the decision-maker should be willing to pay up to $509 in additional monitoring costs to analyze the vapor intrusion potential before making a decision. At best, $509 could pay for two to three passive samples, which in all likelihood would be insufficient to characterize temporal variability in vapor intrusion concentrations sufficiently to obtain a sufficiently low false negative rate. Therefore, in the absence of vast decreases in indoor air sampling costs, remediation would be the preferred alternative for this home.

**Figure 4:**
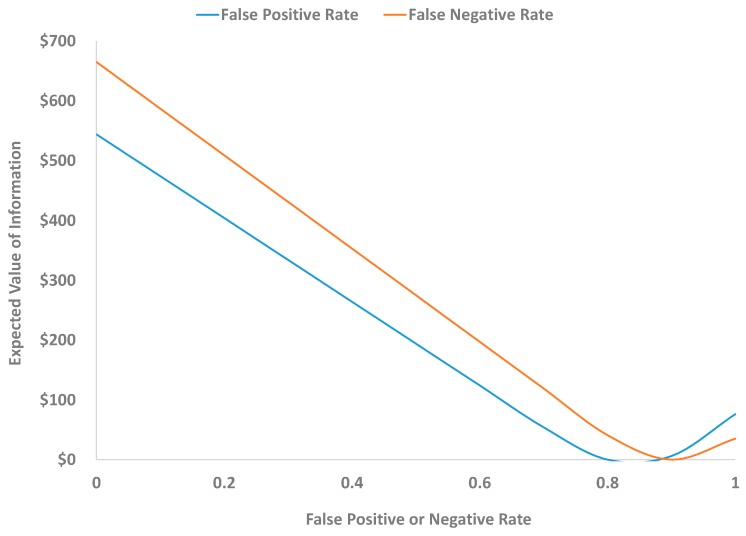
Sensitivity of the expected value of information for the hypothetical example in Figure 2 and Figure 3 to the false positive and false negative rates of the vapor intrusion monitoring program. The value of information declines as the false negative and false positive rates increase (up to the point that the tests are so inaccurate that a negative test suggests that vapor intrusion is occurring and a positive test suggests it is not occurring).

**Figure 5:**
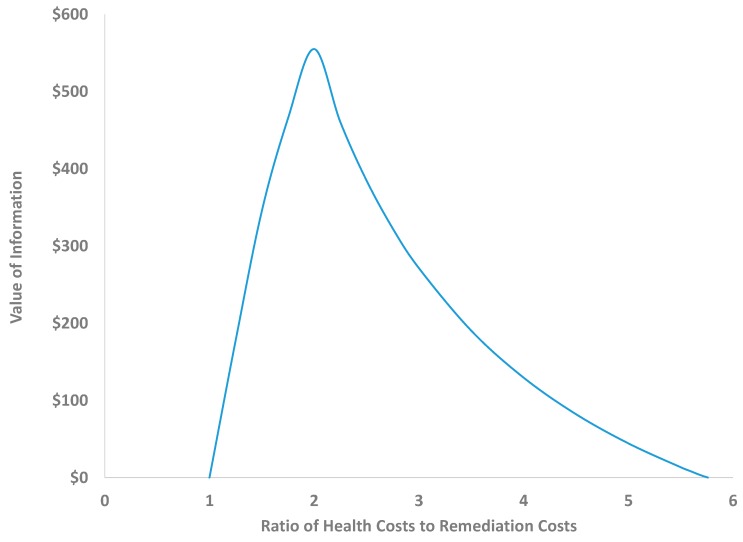
Sensitivity of the expected value of information for the hypothetical example to the ratio of expected health costs in comparison to remediation costs. As this ratio increases beyond 2, the expected value of information decreases as the best decision in the face of high expected health costs is to install the remediation system.

The value of information depends on the multiple uncertain variables illustrated in this example: the prior belief that vapor intrusion is occurring, the accuracy of the test, the expected health costs, and the remediation costs. The effects of all of these variables can be analyzed formally to help further inform remediation decisions. As an example, Figure 4 shows the effects on the value of information when either the false positive or false negative rate changes (keeping all other variables unchanged). As shown, decreasing either rate steeply increases the value of information, with greater benefits coming in this case from decreasing the false negative rate. The value of information is also highly sensitive to the relative costs of potential adverse health effects as compared to remediation costs. Figure 5 shows this effect for the example. When the expected health costs are much larger than remediation costs (the ratio of health to remediation costs is high), information has no value as the decision should always be to remediate. On the other hand, when expected health costs are very low in comparison to remediation costs, then the decision should be not to remediate, and again information has no value. For this example, information has maximum value ($555) when health costs are twice the remediation costs.

The adequacy of data to make decisions about remediation at vapor intrusion sites is often a key point of contention among interested parties and is exacerbated when evidence is conflicting. The vapor intrusion guidance recommends a weight of evidence approach but does not specify methods for weighing the evidence. Rather, the guidance recommends, “When lines of evidence are not concordant and the weight of evidence does not support a confident decision, it may also be appropriate to collect additional lines of evidence, possibly including additional samples.” The value of information approach can be used to help assess whether and how much to invest in additional evidence collection. If collecting additional evidence is highly unlikely to alter the decision about remediation (for example, when health costs are very high or very low in comparison to remediation costs), then information has little to no value. On the other hand, additional information can help resolve or eliminate uncertainties, leading to a decision that may be more acceptable to multiple involved stakeholders. The hypothetical example illustrated here could be easily scaled to a community by adjusting the health and remediation cost information accordingly (for example, by using remediation and health costs for the full community instead of for a single house), in order to support community-wide dialogue about whether to gather more data before making remediation decisions.

## 6. Engaging the Community in Site Assessment and Decision-Making

Concern among residents at vapor intrusion sites is likely to be heightened relative to other kinds of contaminated sites due to the residents’ perceived loss of control over the quality of their indoor living space [55,56]. The complexity and lack of clear science and regulation is an additional source of frustration for community members [57]. To date, community involvement has played a minor role in vapor intrusion exposure assessment or decision-making. While the EPA guidance acknowledges the importance of community involvement, its emphasis is a one-way communication of information from regulators (or scientists) to residents. The current community involvement strategy by the EPA does little to create mechanisms to integrate local knowledge or encourage proactive lay-professional collaborations to examine vapor intrusion. This approach fails to adapt to current trends, where the affected public, especially environmental justice populations living in close proximity to polluting facilities, are demanding a greater role in researching, describing, and prescribing solutions [58].

At contaminated sites, included vapor intrusion sites, there are growing community demands for access to technologies that allow community members to generate their own exposure data and more effectively participate in environmental decision-making [59]. Rather than considering the residents merely the subject of a risk assessment, community members can be equally expert in gathering information about hazards in their own communities and can add new dimensions of knowledge that outside scientists do not have [60,61,62].

In a community-driven (as opposed to one-way communication) approach, the community plays a central role in defining the problems and designing data collection, supplying local knowledge and interpreting results in the context of the local reality [63]. For example, the community can offer insights about which buildings to prioritize and how to approach residents. The involvement of scientists with communities seems a useful and important approach to share expertise, explain what research can and cannot do, and to advise on appropriate methodologies. The community can highlight and safeguard specialized knowledge of the community about the local environment, exposure and activity patterns [64]. Community-driven methods have improved the relationship between scientists and residents enhanced both the quantity and quality of data collected [65,66]. This approach can further facilitate communication with regulators and provide an evidence base for policy setting.

Vapor intrusion assessments need processes for collecting and documenting community knowledge of vapor intrusion exposure and for integrating the results into the multiple lines of evidence approach. This can happen in a facilitated dialogue, whereby the community presents the problems, questions and needs to the regulators. Based on this input, the design to assess the vapor intrusion pathway and the conceptual site models can develop based on a continuous feedback loop with the community. Interactive knowledge-making has the potential to improve community trust in the accountability and credibility of scientific assessments [67].

Furthermore, assessing indoor air requires direct access to the homes of residents. Experience at other sites has suggested that access to measuring indoor air is a barrier to data collection [57]. A small project has demonstrated a passive monitoring technology that community members (after participating in a training) can deploy themselves [13] to collect indoor air samples. The short training includes where to place the monitor, how to deploy it, what information to record (start/stop time, activity data) and and how to package the device after monitoring. We believe this design framework, the simplicity of the sampling devices and integration of local organizations facilitated the adherence of participants to the study protocol in this case. Passive sampling techniques coupled with community partnerships provide a less intrusive and more cooperative approach when compared to using active sample collection canisters deployed without community involvement.

Vapor intrusion exposures may also be influenced by demographic patterns and pose environmental justice concerns—that is, a disproportionate impact on people of color and low-income communities. Indoor air exposures are influenced by factors related to socioeconomics such as dwelling size or housing conditions as well as neighborhood characteristics, such as ambient pollution [68]. Low-income homes are more often smaller, “leakier” and located near pollution sources, including hazardous waste sites [69,70]. People of color communities are also more likely to be burdened multiple environmental health risks [71,72,73]. In addition, when combined with social, economic, and psychological stressors, vapor intrusion exposures may increase vulnerability at the population level [74]. At the same time, these communities are less likely to receive health-protective remediation at hazardous waste sites [75].

In summary, the use of community-based exposure assessment techniques should be expanded. Such techniques employ the community in defining the problems and the necessary data, supplying local knowledge, and interpreting the results in the context of the local reality. Providing resources for independent scientists to work directly with the community and in tandem with agency or industry scientists would improve the perceived credibility of the findings by the community [55,57]. Risk should be considered in light of specific conditions of such communities, including environmental justice concerns.

## 7. Conclusions

### Improving Future Vapor Intrusion Decisions

Based on current evidence, we recommend methods to improve monitoring, modeling and integrating information to support remediation decisions at vapor intrusion sites, building on EPA’s new guidance document. Specifically, we recommend the following step-wise decision-making approach:

(1). Engage the community to target a subset of homes for initial monitoring to confirm if a vapor intrusion pathway is present. With the community, decide whether to use the controlled pressure method to evaluate existence or non-existence of the pathway in the selected homes or proceed directly to step 2.
(2). In the subset of homes where the controlled pressure method was used and revealed the existence of a complete pathway, or in all homes from step 1, engage the community in deploying passive samplers to collect time-integrated indoor air samples over a three-week period during the season in which vapor intrusion is expected to yield the highest concentrations. If sampling in natural conditions, multiple samples per home should be collected in different seasons.
(3). Using the measured indoor air concentrations from step 2, calibrate a stochastic version of the Johnson-Ettinger model as explained in Johnston *et al.*, 2014. Use the upper 95th percentiles of the concentrations predicted in each home as an estimate of long-term average concentration to conduct a house-by-house risk assessment. Identify all homes where this 95th percentile predicted concentration exceeds health-based standards.
(4). For all homes identified in step 3, engage the community to decide whether to install remediation without further monitoring or to conduct further monitoring to confirm the presence and magnitude of vapor intrusion before making a decision. Consider using a value-of-information analysis to help inform these decisions. Consider environmental justice implications of decisions about whether to remediate the homes. That is, remediation decisions should not be based exclusively on cost-benefit analysis but also should consider equity issues.

Exposure assessment is a key component of decision-making, but nonetheless just one component to understanding the ecological, human health, economic and legal implications of diminished indoor air quality. Future analysis of vapor intrusion guidance and regulation approaches should further consider: (a) who bears the burden of proof; (b) the evidence required to pass a confidence threshold; and (c) co-benefits, that is the effects that are favorable to human welfare that are not directly related to the benefit of vapor intrusion mitigation. This integrative context would equip decision-makers and affected residents with additional critical information to assess vapor intrusion and make remediation decisions.
